# Transsynaptic degeneration of ventral horn motor neurons exists but plays a minor role in lower motor system dysfunction in acute ischemic rats

**DOI:** 10.1371/journal.pone.0298006

**Published:** 2024-04-26

**Authors:** Lei Zhang, Jingwen Liu, Mingsheng Liu

**Affiliations:** Department of Neurology, Peking Union Medical College Hospital, Chinese Academy of Medical Sciences, Beijing, China; Wright State University, UNITED STATES

## Abstract

**Background:**

As a leading cause of mortality and long-term disability, acute ischemic stroke can produce far-reaching pathophysiological consequences. Accumulating evidence has demonstrated abnormalities in the lower motor system following stroke, while the existence of Transsynaptic degeneration of contralateral spinal cord ventral horn (VH) neurons is still debated.

**Methods:**

Using a rat model of acute ischemic stroke, we analyzed spinal cord VH neuron counts contralaterally and ipsilaterally after stroke with immunofluorescence staining. Furthermore, we estimated the overall lower motor unit abnormalities after stroke by simultaneously measuring the modified neurological severity score (mNSS), compound muscle action potential (CMAP) amplitude, repetitive nerve stimulation (RNS), spinal cord VH neuron counts, and the corresponding muscle fiber morphology. The activation status of microglia and extracellular signal-regulated kinase 1/2 (ERK 1/2) in the spinal cord VH was also assessed.

**Results:**

At 7 days after stroke, the contralateral CMAP amplitudes declined to a nadir indicating lower motor function damage, and significant muscle disuse atrophy was observed on the same side; meanwhile, the VH neurons remained intact. At 14 days after focal stroke, lower motor function recovered with alleviated muscle disuse atrophy, while transsynaptic degeneration occurred on the contralateral side with elevated activation of ERK 1/2, along with the occurrence of neurogenic muscle atrophy. No apparent decrement of CMAP amplitude was observed with RNS during the whole experimental process.

**Conclusions:**

This study offered an overview of changes in the lower motor system in experimental ischemic rats. We demonstrated that transsynaptic degeneration of contralateral VH neurons occurred when lower motor function significantly recovered, which indicated the minor role of transsynaptic degeneration in lower motor dysfunction during the acute and subacute phases of focal ischemic stroke.

## Introduction

Acute ischemic stroke, a principal cause of mortality and long-term disability, produces immense health and economic burdens globally [1]. In recent years, a growing number of studies have focused on the far-reaching pathophysiological consequences of stroke, such as impaired motor pathways, autonomic dysfunction and peripheral immunodepression [2]. Among these sequelae, motor pathway impairment is the major factor contributing to functional disability. Therefore, a better understanding of the physiological and pathological changes in the motor pathway after stroke is necessary, which is key to identifying suitable neuromodulation therapies and rehabilitation strategies.

The term “transsynaptic degeneration” is used to describe the phenomenon that secondary neurodegeneration could occur in remote regions from the primary site of damage, spreading between directly anatomically connected neurons [3–8], or between not directly connected neurons [9] in pathological conditions, including cerebral infarction, neurodegenerative diseases and retinal diseases. The existence of transsynaptic degeneration of contralateral spinal ventral horn (VH) neurons following an ischemic stroke is still in dispute [10–14], although motor unit loss following stroke has been reported and confirmed by previous clinical studies [13, 15–18] and animal experiments [19, 20].

The mechanisms driving transsynaptic degeneration have not yet been thoroughly elucidated. Improper activation of the inflammatory response, neurotoxic factors, oxidative damage and apoptosis were proposed to play a role in triggering this devastating progression [6, 21–24]. During inflammatory response in the central nervous system, microglia are important innate immune cells, and are the first cell type to respond to insults. Wu and colleagues observed recruitment and activation of microglia in both the contralateral dorsal and ventral horns of the lumbar spinal cord in rats after permanent occlusion of the middle cerebral artery (MCA) [22]. Work performed by Hosp and colleagues revealed a mild inflammatory response in the exofocal area where dopaminergic neurodegeneration was detected following motor-cortical stroke, suggesting inflammation was not driving neurodegeneration [9]. Hence, more work is required to determine the role of microglia in transsynaptic degeneration. Moreover, increasing evidence has indicated a deleterious effect of extracellular signal-regulated kinase (ERK) pathway activation in pathological conditions such as neurodegenerative diseases and spinal cord injury [25–28], while little is known about the role of ERK pathway activation in transsynaptic degeneration.

Herein, using a rat model of acute ischemic stroke, we aimed to investigate the overall pathological and physiological changes in the lower motor unit over time after stroke by measuring the modified neurological severity score (mNSS), compound muscle action potential (CMAP) amplitude, repetitive nerve stimulation (RNS), spinal cord VH neuron counts, and the corresponding muscle fiber morphology simultaneously, which was different from previous studies where researchers usually focused on abnormalities in one anatomically isolated region. In addition, the activation statuses of microglia and ERK1/2 were assessed to preliminarily elucidate the mechanism underlying neuronal transsynaptic degeneration.

## Materials and methods

### Animals

Adult male Sprague‑Dawley rats were obtained from the Animal Center of Peking Union Medical College Hospital (PUMCH) (n = 49, 300–380 g). We abided by the NIH guidelines for the care and use of laboratory animals (8th edition, NIH), and all animal experiments were approved by the Ethics Committee of the PUMCH (license number: XHDW-2021-031). Research staff were trained by Beijing Association on Laboratory Animal Care. All animals were taken good care of, housed in cages with a 12/12-h light/dark cycle at room temperature, and had access to food and water ad libitum. Before surgery and electrophysiological examinations, rats were deeply anesthetized with 2% isoflurane. During the operation, the respiration rate was monitored, and the body temperature was maintained at 37°C using a heated pad. After surgery, animals received a subcutaneous injection of buprenorphine for analgesia (0.02 mg/kg). The overall health and incision healing status of each rat were closely observed daily. All efforts were made to minimize suffering.

Rats were randomly assigned to two groups: (1) 44 rats were subjected to the MCA occlusion (MCAO) method of ischemic stroke. Seven rats died during the perioperative period, because of severe brain edema or subarachnoid hemorrhage (the autopsy confirmed). Twelve rats were excluded for severe functional deficits (mNSS ≥ 7) at 1 day post stroke because they had problems eating and drinking and a minority of these rats survived 14 days post stroke. Fifteen rats were excluded for minor functional deficits (mNSS ≤ 2). Ultimately, 10 rats were included in the MCAO groups, 5 of which were sacrificed on day 7 after stroke (MCAO-7d group, n = 5), and the other 5 rats were sacrificed on day 14 after stroke (MCAO-14d group, n = 5). (2) Five rats were subjected to sham surgery (control group, n = 5).

In total, 27 rats were euthanized by isoflurane overdose according to mNSS score (mentioned above) immediately after the first assessment of post-stroke deficits (1 day after MCAO). The other 15 rats used for experiments were sacrificed on day 7 post stroke, on day 14 post stroke, or on day 14 post sham surgery by isoflurane overdose.

Investigators performing experimental assessments were blinded to the group assignment.

### Induction of permanent focal ischemic stroke

Permanent focal cerebral ischemia was induced as described previously [29]. Briefly, anesthetized animals were laid on a warm operation table, and the right common carotid artery (CCA), the internal carotid artery (ICA), and the external carotid artery (ECA) were exposed. Then, the ECA was ligated, and the CCA and the ICA were temporarily clipped with a vessel clip. A monofilament nylon suture (diameter = 0.40 mm; Beijing CinonTech, China) was then inserted into the ICA, the vessel clip on the ICA was removed, and then the suture was gently advanced until resistance was felt, indicating that the tip of the suture had reached the proximal segment of the anterior cerebral artery (ACA). This nylon suture was left in place until sacrifice. Rats in the control group received sham surgeries in which only the CCA, ICA, and ECA were exposed.

As we noted, nylon sutures without any coating materials obtained from Beijing CinonTech were used for MCAO model establishment, which had a round tip and a thick wire (diameter ratio: 1.5/1). The manufacturer recommended this kind of suture to induce a permanent MCAO model. After surgery, the blood flow from the ACA was entirely blocked, while a small stream of blood flow might run through the remaining lumen of the ICA into the MCA. The features of the nylon sutures used in this study might lead to variability in the neurological deficit scale, while enabling most of the MCAO rats to exhibit a mild to moderate neurological deficit and survive longer.

### Assessment of post-stroke deficits

We employed the widely used and accurate scale, the modified neurologic severity score (mNSS) [32], to evaluate the post-stroke neurological functional deficits of animals. This scale was composed of tests on motor, sensory, reflex, balance and abnormal movements with scores ranging from 0 to 18 (normal score, 0; maximal deficit score, 18) (details of how mNSS scoring was performed were shown in S1 Table, referring to the work of Chen et al. [30]).

In vivo longitudinal evaluations were carried out at baseline, and were repeated at 1 day, 7 days, and 14 days after stroke. Rats with severe functional deficits (mNSS ≥ 7) at 1 day post stroke were excluded because they had problem eating and drinking, and a minority of these rats survived 14 days post stroke. Rats with very slight deficits (mNSS ≤ 2) were excluded as well.

### Electrophysiological assessment

CMAP amplitudes were recorded from the bilateral gastrocnemius after stimulation of the sciatic nerve using a portable electromyography (EMG) machine (10CH Medelec Synergy, Natus Europe GmbH, Germany) as previously reported by Lin [19] with surface stimulating and recording electrodes (to avoid muscle injury). Briefly, under anesthesia (2% isoflurane), the sciatic nerve was stimulated at the root of the hindlimb, and the surface stimulating electrode was placed at the anterior superior iliac spine. CMAPs were recorded by surface electrodes on the gastrocnemius, and CMAP amplitudes were measured from onset to the negative peak. For the RNS test, at least 10 stimuli with a low frequency of 5 HZ were performed. Decrements of the amplitudes from the first to fifth CMAPs were recorded, and a decline proportion of at least 15% was regarded as abnormal. To evaluate the overall motor function of the spinal cord and peripheral nervous system, CMAP amplitudes and RNS were recorded at baseline and were repeated at 1 day, 7 days, and 14 days after stroke.

### Tissue preparation

Rats were sacrificed on day 7 post stroke, on day 14 post stroke, or on day 14 post sham surgery by isoflurane overdose. Animals were perfused transcardially with 4% paraformaldehyde (PFA). Then the brain, L4-L5 spinal cord, and gastrocnemius muscles were removed quickly. These tissues were kept in 4% PFA for 24 h before being embedded in paraffin, and then cut into slices coronally (the brain) or transversely (the spinal cord and gastrocnemius muscles).

### Hematoxylin and eosin (H&E) staining

The 10-μm-thick sections of the brain, the L4-L5 spinal cord, and the gastrocnemius muscle were used for H&E staining. Briefly, sections were deparaffinized, rehydrated, and stained with hematoxylin for 3 min. Then sections were washed with running water for 15 min, and stained in eosin for 15 min followed by washing in a 70, 80, 90, 95, and 100% EtOH series. Finally, the sections were washed twice in xylene and then examined by light microscopy (Nikon Eclipse E100). Muscle fiber diameters were measured in photomicrographs of H&E-stained muscle tissue sections with ImageJ software. Fifty myofibers per condition were counted (muscle fibers with possible neurogenic atrophy, shrinkage in size and angular appearance were excluded).

### Immunofluorescent labeling

The 3-μm-thick sections of the lower lumbar spinal cord were utilized for immunofluorescent labeling. Briefly, sections were deparaffinized through a standard procedure and blocked with 1% BSA in phosphate-buffered saline with 3% Triton-X-100. Sections were then incubated with primary antibody solution at 4°C overnight and then incubated with secondary antibodies for 2 h at room temperature. Sections were mounted and examined under a fluorescence microscope (Nikon Eclipse C1). The primary antibodies included rabbit polyclonal anti-neurofilament-200 (anti-NF-200) (1:200; 18934-1-AP, ProteinTech, China), mouse monoclonal anti-phospho-ERK1/2 (1:50; 5726S, CST, USA), and rabbit monoclonal anti-Iba-1 (1:100; ab178846, Abcam, MA). The secondary antibodies included CoraLite488-conjugated Affinipure Goat anti-rabbit IgG (H+L) (1:100; SA00013-2, ProteinTech, China) and CoraLite594-conjugated goat anti-mouse IgG(H+L) (1:200; SA00013-3, ProteinTech, China).

The VH area was defined as the area ventral to the horizontal line passing through the central canal. VH motor neurons can be identified based on staining of neuron-specific markers (such as Nissl substance, NeuN, SMI-32, or NF-200) along with morphological criteria of large size, multipolar and obvious nuclei [8, 12, 31–35]. In this study, only NF-200-positive VH horn cells with a distinct nucleus and a soma diameter of ≥ 25 μm were counted as lower motor neurons. Microglia were defined as iba-1-positive cells with DAPI-positive nuclei, and were counted bilaterally. NF-200-positive VH cells coexpressing p-ERK1/2 were also counted.

### Statistical analysis

The software IBM SPSS Statistics, version 22, was employed for statistical analysis. All data were processed to verify the normality test for variables. If the normality test failed, the data were analyzed with non-parametric test. If the normality test passed, we conducted statistical analysis with one-way analysis of variance (one-way ANOVA) followed by the Student-Newman-Keuls test (homogeneity of variance) or Dunnett’s T3 test (heterogeneity of variance) to compare independent variables (the pathological findings on the paretic side among various groups). The mNSS progression between different time points was compared using the Wilcoxon matched-pair signed ranks test. The electrophysiological results at different time points, as well as the pathologic results between bilateral sides at one time point, were compared using paired t‑tests. Throughout the results section, data is listed as mean ± standard deviation. *p* < 0.05 was employed to delineate significance for analysis of all results.

### Ethical publication statement

We confirm that we have read the Journal’s position on issues involved in ethical publication and affirm that this report is consistent with those guidelines.

## Results

### MCAO rats exhibited neurologic deficits that recovered with time

**Fig 1:**
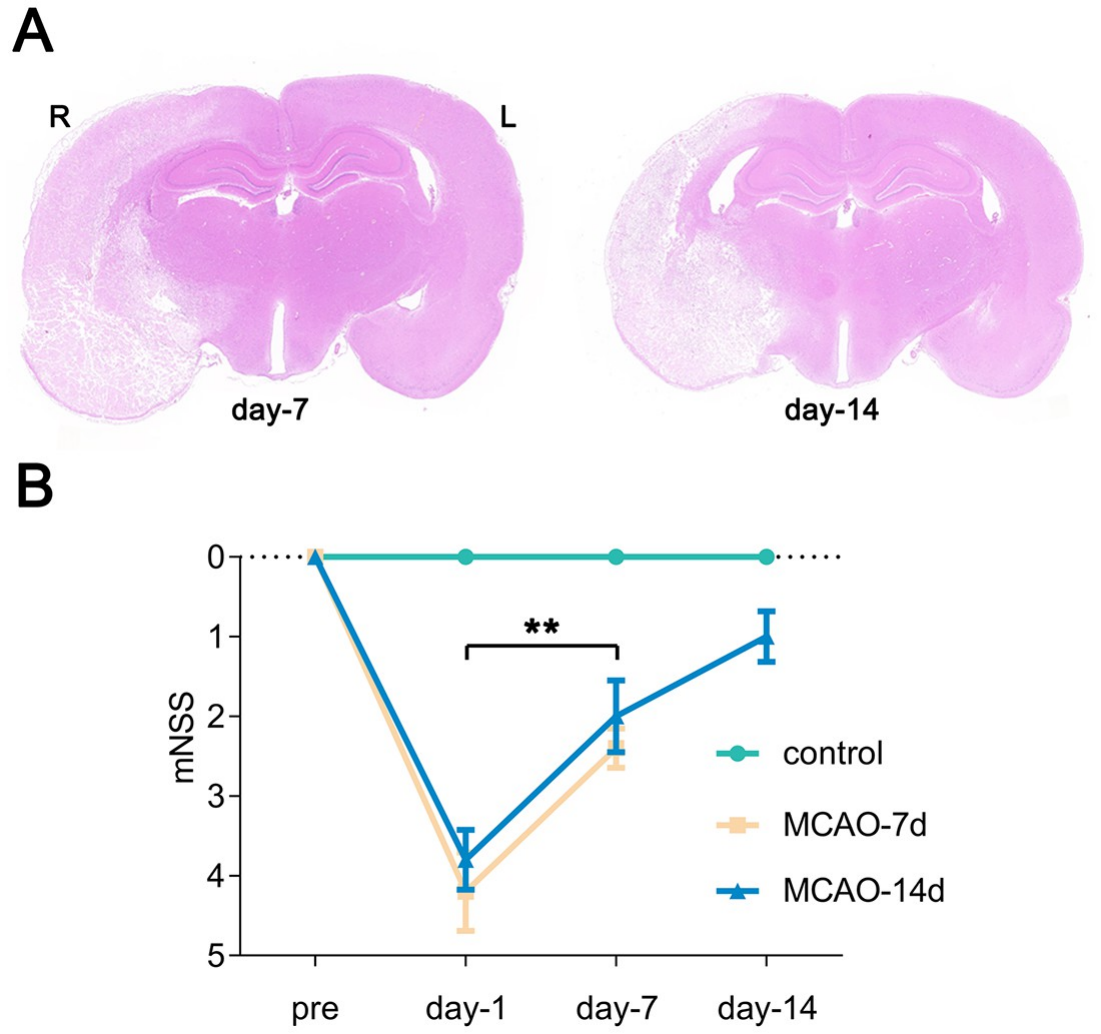
Evaluation of neurological deficits in control and MCAO rats. Panel A: Images indicating the extent of a focal ischemic lesion in the brain. Panel B: Neurological deficits evaluated by the mNSS attenuated with time after surgery. At baseline, day 1 and day 7, n = 5 in the MCAO-7d group, n = 5 in the MCAO-14d group, n = 5 in the control group; on day 14, n = 5 in the MCAO group, n = 5 in the control group. Bar graphs with error bars represent the mean ± SD. The Wilcoxon matched-pair signed rank test was used. In the MCAO group, day-1 vs. day-7, **p < 0.01; day-7 vs. day-14, *p* = 0.059.

To roughly display the successful establishment of MCAO models, H&E staining of coronal brain sections were conducted for pathological assessment. As shown in Fig 1A, the brain tissue ipsilateral to MCAO appeared loose and lightly stained, with enlarged interstitial spaces, compared to the contralateral half of the brain. At 7 days after MCAO, the ipsilateral cortex was infarcted with obvious edema, and the ipsilateral lateral ventricle disappeared in this section, while at 14 days after surgery, brain edema was significantly relieved. The neurological functional deficits were estimated using the mNSS at 1 day, 7 days, and 14 days after MCAO or sham surgery, as shown in Fig 1B. The mNSS peaked at 1 day after surgery in MCAO rats (n = 10, 4.0 ± 0.9) and was significantly reduced by 45% at 7 days (n = 10, 2.2 ± 0.6) (*p* < 0.01), indicating a distinct functional recovery. At 14 days post-MCAO, neurological deficits were recovering but the mNSS scores were still trending increased compared to controls (n = 5, 1.0 ± 0.5). The deficits related to hindlimb function could be estimated by flexion of the hindlimb and the walking test in the mNSS assessment, and at least 1 point was awarded in MCAO rats at 1 day after surgery. The function of the hindlimb partially recovered as estimated by the motor section of the mNSS (scored 0–1 in flexion of the hindlimb and the walking test at 14 days after surgery). Additionally, flexion of the forelimb was the most common deficit observed at 14 days post MCAO. No changes were observed in the scores of rats in the sham group at different time points (0 points).

### CMAP amplitudes decreased in the contralateral hindlimb after focal cerebral ischemic stroke

**Fig 2:**
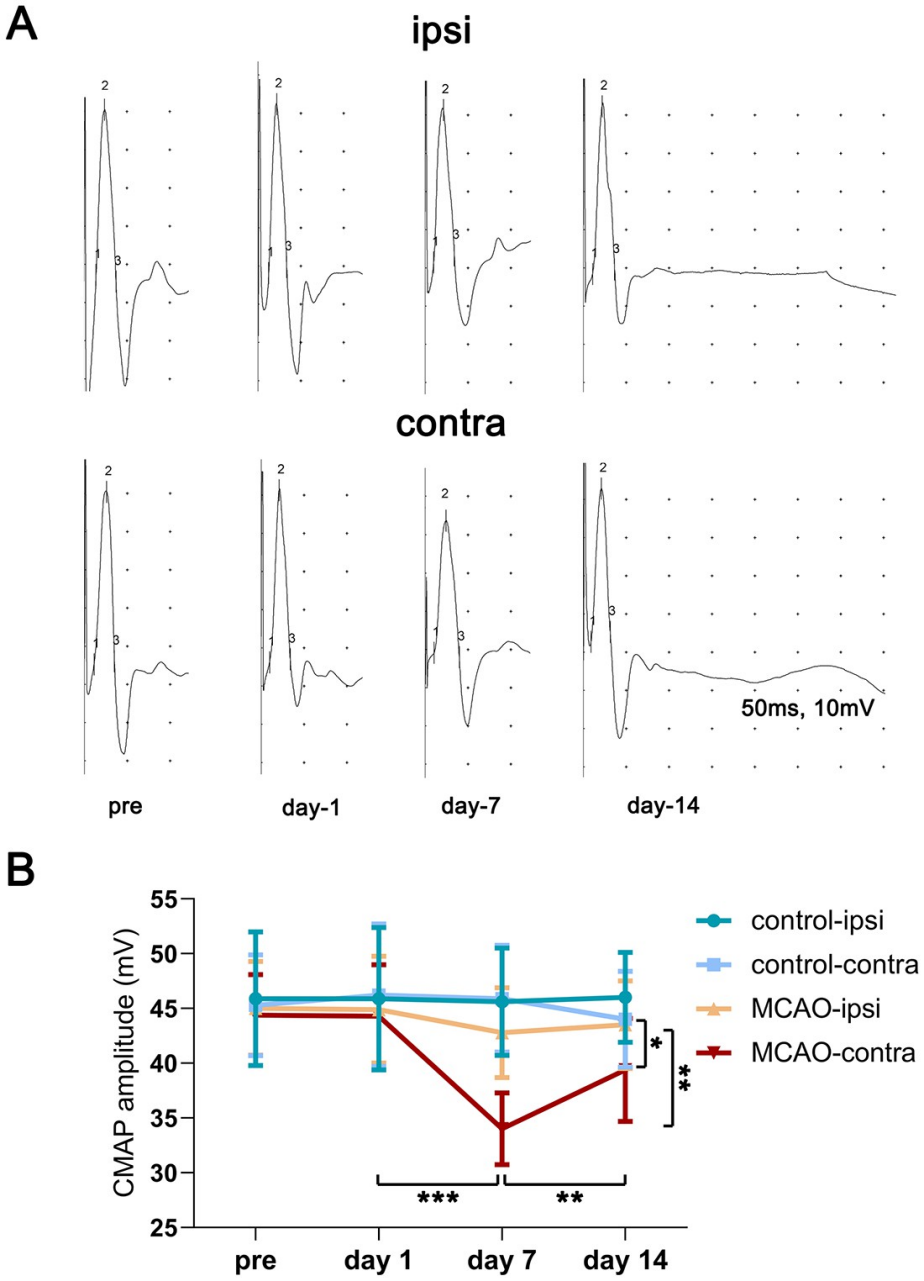
Detection of CMAP amplitudes in control and MCAO rats. Panel A: Representative images of CMAPs in one MCAO rat in the MCAO-14 group. The numbers “1”, “2” and “3” indicate the onset, the negative peak and the end of the negative wave of the CMAP, respectively. CMAP amplitude was measured from onset to the negative peak. Panel B: Data represent variables of CMAP amplitudes quantified from the contralateral and ipsilateral hindlimbs after focal ischemic injury or sham surgery. At baseline, day-1 and day-7, n = 10 in the MCAO group, n = 5 in the control group; on day 14, n = 5 in the MCAO group, n = 5 in the control group. Bar graphs with error bars represent the mean ± SD. A paired t‑test was employed in the statistical analysis. Day-1 vs. day-7 in the contralateral side of MCAO rats, ***p < 0.001. Day-7 vs. day-14 in the contralateral side of MCAO rats, **p < 0.01. Contralateral vs. ipsilateral on day-7 in MCAO rats, **p < 0.01. Contralateral vs. ipsilateral on day-14 in the same rat, *p < 0.05.

The abnormalities in CMAPs could reflect integral impairments in the lower motor unit, including impairments in VH neurons, the motor fibers of peripheral nerves, neuromuscular junctions and skeletal muscles. Hence, we carried out motor nerve conduction studies to record CMAPs at baseline, and repeated them at 1 day, 7 days, and 14 days after MCAO or sham surgeries. As exhibited in Figs 1 and 2, MCAO rats experienced the most severe neurological functional disability at 1 day post stroke, and recovered with time. In contrast, CMAP amplitudes of the paretic limbs were significantly reduced and reached a minimum at 7 days after stroke (n = 10, at baseline = 44.4 ± 3.7 mV, day-1 = 44.3 ± 4.7 mV, day-7 = 34.0 ± 3.3 mV; n = 5, day-14 = 39.5 ± 4.7 mV; day-1 vs. day-7, *p* < 0.001). The CMAP amplitudes of the unaffected limbs exhibited a slight reduction on day 7 in MCAO rats, compared with the day-1 values, but the difference was not significant (n = 10, at baseline = 45.0 ± 4.25 mV, day-1 = 44.9 ± 4.9 mV, day-7 = 42.8 ± 4.1 mV; n = 5, day-14 = 43.5 ± 4.0 mV; day-1 vs. day-7, *p* = 0.146). In the control group, CMAP amplitudes at different time points were not significantly different.

### Transsynaptic degeneration in the contralateral VH occurred in the subacute phase of ischemic stroke

**Fig 3:**
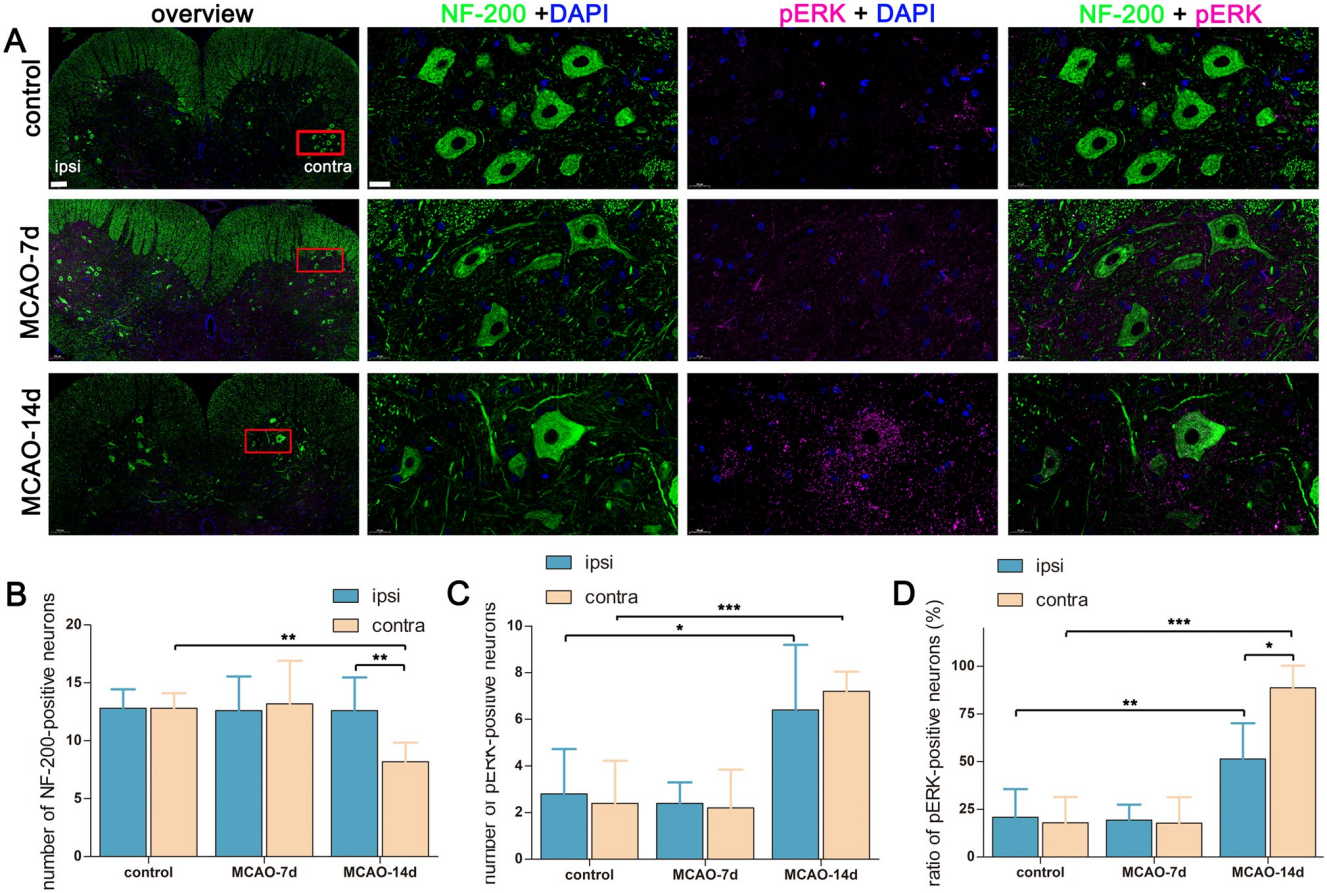
Labeling the spinal cord with anti-NF-200 and anti-p-ERK antibodies. Panel A: Representative images of 5 independent experiments taken from the L5 spinal cord. For the overview figures, scale bar = 100 μm. For the insight figures, scale bar = 20 μm. Panel B: Quantifying VH neuron numbers contralaterally and ipsilaterally. n = 5 for each group. Bar graphs with error bars represent the mean ± SD. On the contralesional side, control group vs. day-14, **p < 0.01; control group vs. day-7, *p* = 0.993; day-7 vs. day-14, *p* = 0.092. On day-14 post-MCAO, contralateral vs. ipsilateral, **p < 0.01. Panel C: Quantifying p-ERK-positive VH neuron numbers contralaterally and ipsilaterally. n = 5 for each group. Bar graphs with error bars represent the mean ± SD. On the contralesional side, control group vs. day-14, ***p < 0.001; day-7 vs. day-14, ***p < 0.001. On the ipsilesional side, control group vs. day-14, *p < 0.05; day-7 vs. day-14, *p < 0.05. On day 14 post-MCAO, contralateral vs. ipsilateral, *p* = 0.512. Panel D: Quantification of the proportion of p-ERK-positive VH neurons contralaterally and ipsilaterally. On the contralesional side, control group vs. day-14, ***p < 0.001; day-7 vs. day-14, ***p < 0.001. On the ipsilesional side, control group vs. day-14, **p < 0.01; day-7 vs. day-14, **p < 0.01. On day 14 post-MCAO, contralateral vs. ipsilateral, *p < 0.05. In this section, one-way ANOVA was used to compare between groups, while a paired t‑test was used to compare between the contralateral side and the ipsilateral side.

To evaluate whether transsynaptic degeneration of VH neurons existed, we recorded the motor neuron number in the contralesional VH, and compared it with that in the ipsilesional VH in MCAO rats, or compared it with that in the contralateral VH in the sham surgery group. The contralesional VH neuron number in the MCAO-7d group was not significantly different from that in the control group, while there was a significant difference in contralesional VH neuron numbers between the MCAO-14d group and the control group (n = 5 in each group, in the contralateral VH, control = 12.8 ± 1.3, MCAO-7d = 13.2 ± 3.7, MCAO-14d = 8.2 ± 1.6. Control vs. MCAO-14d, *p* < 0.01.). Comparisons between the ipsi- and contralesional regions in the lower lumbar spinal cord revealed a selective loss of NF-200-positive neurons on the contralateral side at 14 days post-stroke (n = 5, ipsilesional = 12.6 ± 2.9, contralesional = 8.2 ± 2.7, *p* < 0.01), as shown in Fig 3A and 3B. Moreover, at 14 days post-MCAO, upregulated activation of ERK1/2 occurred in the bilateral VH, and positive p-ERK immunoreactivity was mainly located in the cytoplasm of the VH motor neurons (n = 5, the number of p-ERK-positive neurons in the ipsilesional VH = 6.4 ± 2.8, the number of p-ERK-positive neurons in the contralesional VH = 7.2 ± 0.8, *p* = 0.512) (Fig 3A and 3C). The proportion of p-ERK-positive VH neurons significantly increased in the contralateral side (n = 5, 88.7 ± 11.6%), compared to that in the ipsilateral side in the same MCAO rat (n = 5, 51.4 ± 18.7%, *p* < 0.05), or compared to that in the contralateral side in the control group (n = 5, 18.0 ± 13.4%, *p* < 0.001) (Fig 3A and 3D).

### Microglial recruitment and activation in the contralateral VH appeared at the early time post stroke

**Fig 4:**
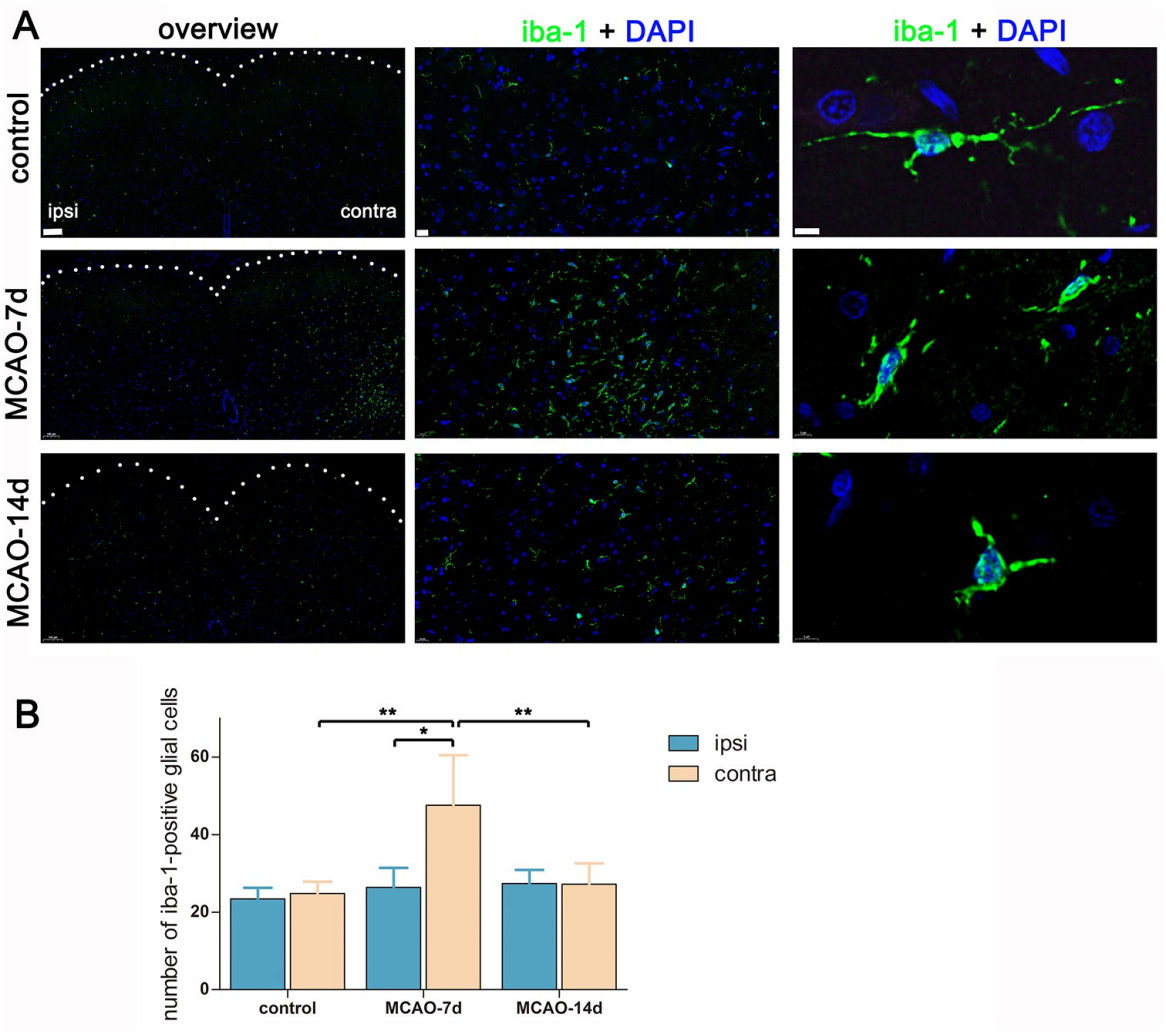
Labeling the spinal cord with anti-iba-1. Panel A: Representative images taken from the L5 spinal cord. For the overview figures, scale bar = 100 μm. For the insight figures exhibiting iba-1 immunoreactivity in the contralateral VH, scale bar = 20 μm. For the insight figures showing the morphology of microglial cells, scale bar = 5 μm. Panel B: Quantifying iba-1-positive microglial cell numbers contralaterally and ipsilaterally in the VH. n = 5 for each group. Bar graphs with error bars represent the mean ± SD. One-way ANOVA was used to compare between groups, while a paired t‑test was used to compare the contralateral side and the ipsilateral side. On the contralesional side, control vs. MCAO-7d, **p < 0.01; MCAO-7d vs. MCAO-14d, **p < 0.01. On day-7 post-MCAO, contralateral vs. ipsilateral, *p < 0.05.

To estimate the status of neuroinflammation, and to superficially evaluate its role in the occurrence of transsynaptic degeneration, we labelled spinal cord microglial cells with an anti-iba-1 antibody, counted the number of iba-1-positive cells and observed their morphology. On day 7 post MCAO, microglial cells recruited to the contralateral VH, leaving the ipsilateral VH almost intact ((n = 5, ipsilesional = 26.4 ± 5.0, contralesional = 47.6 ± 12.8, *p* < 0.05) (Fig 4). The recruited microglial cells exhibited a long-rod morphology with reduced branching, indicating an activated status of these cells. On day 14 post MCAO, the upregulated inflammatory response was partially relieved spontaneously with attenuated microglia recruitment (n = 5 in each group, in the contralateral VH, control = 24.8 ± 3.0, MCAO-7d = 47.6 ± 12.8, MCAO- 14d = 27.2 ± 5.4. Control vs. MCAO-7d, *p* < 0.01; MCAO-7d vs. MCAO-14d, *p* < 0.01).

### Gastrocnemius muscle disuse atrophy peaked on day 7 post stroke, while neurogenic muscle atrophy appeared on day 14 post stroke

**Fig 5:**
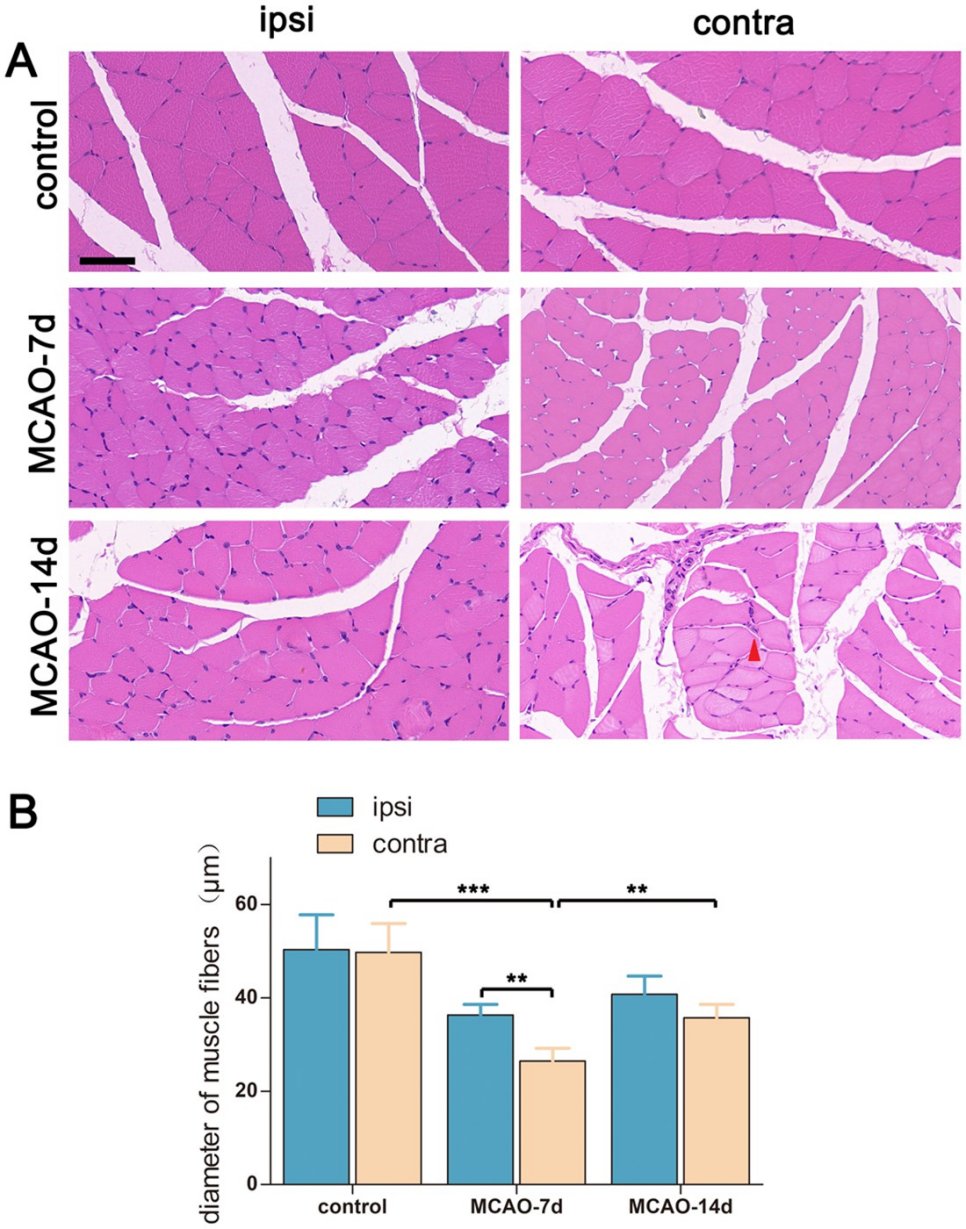
Labeling the gastrocnemius muscle with H&E staining. Panel A: Representative images taken from the bilateral gastrocnemius muscles. The red arrowhead indicates muscle fibers with neurogenic atrophy. Scale bar = 50 μm. Panel B: Quantification of muscle fiber diameters contralaterally and ipsilaterally. n = 5 for each group. Bar graphs with error bars represent the mean ± SD. One-way ANOVA was used to compare between groups, while a paired t-test was used to compare the contralateral side and the ipsilateral side. In the contralateral gastrocnemius muscle, control vs. MCAO-7d, ***p < 0.001; MCAO-7d vs. MCAO-14d, **p < 0.01. On day-7 post-MCAO, contralateral vs. ipsilateral, **p < 0.01.

We explored the muscle fiber morphology bilaterally in each group with H&E staining to assess muscle abnormalities post stroke. To our surprise, the muscle fiber diameters of both hindlimbs decreased to a minimum at 7 days post stroke (n = 5 in each group). In the contralateral gastrocnemius muscle, control = 49.7 ± 6.2 μm, MCAO-7d = 26.4 ± 2.8 μm, MCAO-14d = 35.7 ± 2.9 μm. Control vs. MCAO-7d, *p* < 0.001. In the ipsilateral gastrocnemius muscle, control = 50.3 ± 7.4 μm, MCAO-7d = 36.3 ± 2.3 μm, MCAO-14d = 40.7 ± 3.9 μm. Control vs. MCAO-7d, *p* < 0.01 (Fig 5). On day 7 post stroke, the muscle fibers of the paretic gastrocnemius muscles were significantly smaller than those of the ipsilateral muscles (*p* < 0.01), while no neurogenic muscle atrophy was observed. From 7 days post stroke to 14 days post stroke, the muscle fiber diameters of the contralesional gastrocnemius muscle significantly increased (*p* < 0.01) concurrent with the improved mNSS and increased CMAP amplitudes. The muscle fiber diameters of the ipsilateral gastrocnemius muscle slightly increased, but the difference was not statistically significant (*p* = 0.191). On day 14 post-stroke, muscle fibers with a significantly reduced size and an angular shape (indicating neurogenic muscle atrophy) were observed sporadically in the contralesional gastrocnemius muscle, concurrent with motor neuron loss in the contralesional VH, which might provide additional clear and convincing evidence for transsynaptic degeneration. During the whole subacute phase after stroke, no leukocyte infiltration was observed.

### No obvious abnormalities in neuromuscular junction (NMJ) function were found by RNS after focal stroke

**Fig 6:**
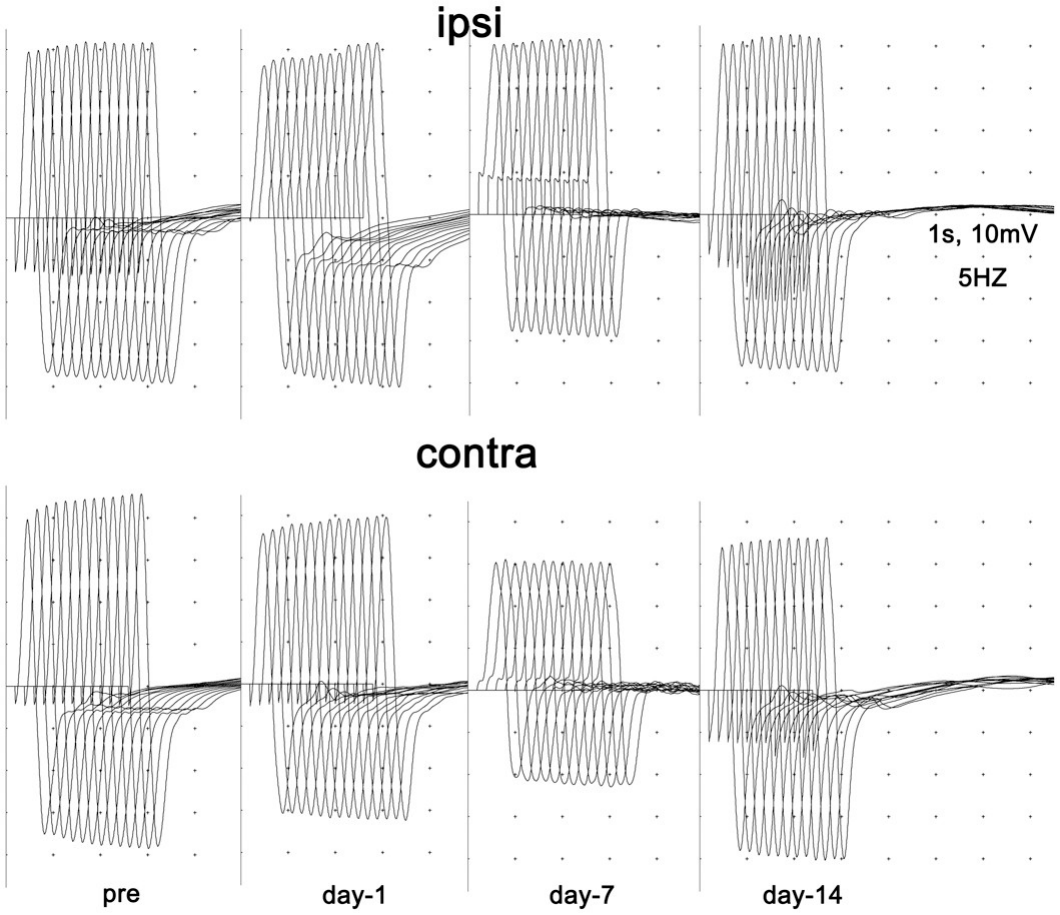
Conducting low frequency RNS examinations bilaterally in control and MCAO rats. Representative images of consecutive low frequency RNS tests (5 Hz) in one MCAO rat at different time points are shown. No obvious decreases in CMAP amplitudes in response to repetitive electrical stimuli were observed at any time point.

Low frequency RNS tests (5 Hz) were performed at baseline and repeated at 1 day, 7 days and 14 days after stroke induction or after sham surgeries (Fig 6). Because moderate-frequency and high-frequency electrical stimuli were reported to exert harmful or beneficial effects on motor axon stability [36], corticospinal plasticity [37, 38] and muscle atrophy [39], high frequency RNS tests were not performed to minimize variables affecting the motor pathways. No apparent decreases in CMAP amplitudes were observed with repetitive electrical stimuli in the bilateral gastrocnemius muscles of rats in all groups.

## Discussion

Motor pathway impairment is common post stroke, and is the leading cause of physical disability [40, 41]. Researchers have made achievements in explaining the underlying mechanisms of impaired lower motor unit function, and transsynaptic degeneration, myofiber atrophy, functional motor unit inactivity, and neuromuscular junction disturbance are thought to play a role in motor function abnormalities [13–15, 19, 20, 42, 43]. Previous reports usually focused on a single component of the lower motor unit, while in this study, we observed the evolution of parameters reflecting several components simultaneously.

In this study, the neurological functional deficiency peaked at 1 day post MCAO surgery, reflected by the rapidly increased mNSS, while at the same time, no obvious lower motor unit deficits were observed (indicated by normal CMAP amplitudes), which indicated that motor abnormalities in the hyperacute phase of ischemic stroke were more likely to be attributed to the upper motor neuron dysfunction.

At 7 days after stroke, the functional deficit partially recovered, as indicated by decreased mNSS, while the CMAP amplitude declined to a nadir, indicating severely injured lower motor unit function. Meanwhile, microglia recruitment and activation were detected on the paretic side (contralateral to the side of the brain lesion) of the spinal cord VH, while there were no significant changes in the structure or the number of VH neurons, which was consistent with the work of Wu [44]. Wu and colleagues reported that 5 days after MCAO, structurally unaltered VH motor neurons were surrounded by microglial cells, while selectively dying dorsal horn neurons were phagocytosed by vigorous microglia cells. Since there was a discrepancy between VH neuron loss and microglia recruitment and activation, we speculated that the activation and recruitment of microglial cells were not the triggers of transsynaptic degeneration and might exert a neuroprotective effect on VH motor neurons. Recent literature supports our viewpoints: (1) Emerging evidence indicates that microglia respond to neuronal hyperactivity by suppressing neuronal activity and promoting homeostasis, and then dampening seizures, while ablation of microglia amplifies the activity of neurons, leading to seizures [45–47]. Moreover, glutamate, the excitatory transmitter of sensorimotor cortex neurons, could induce microglial responses independent of overt neuronal injury [47]. Hence, from our point of view, the release of a large amount of glutamate from the degenerating glutaminergic corticospinal fibers induced microglial responses in the contralateral spinal cord. Then, the activity of postsynaptic VH motor neurons was suppressed, and motor neurons were protected. The suppressed VH neuron activity might partially contribute to the injured lower motor unit function (indicated by decreased CMAP amplitude detected in the contralesional hindlimb) on day 7 post stroke. The resolution of the microglial response from day 7 to day 14 post-stroke might contribute to the increased CMAP amplitude and possibly to the improved overall neurological deficits (reflected by the mNSS). (2) Researchers have demonstrated that microglia can protect neurons in pathological conditions other than epilepsy. Cserép et al. found that the infarct volume was increased alongside a worse neurological outcome after microglia inhibition [48]. Dong Y and colleagues demonstrated that microglial cells could neutralize oxidized phosphatidylcholines (OxPCs) induced neurodegeneration (OxPCs are potent drivers of neurodegeneration found in brain sections of multiple sclerosis patients) [49]. (3) Methylxanthines including pentoxifylline, propentofylline and pentifylline, which attenuated microglial reactions after stroke [50, 51], failed to exert protective effects on patients with acute ischemic stroke [52].

On day 7 after stroke, the muscle fiber diameters of the bilateral gastrocnemius muscles were significantly decreased to the nadir, and the decrement was greater in the paretic hindlimb, which was consistent with previous studies conducted in humans and summarized by English et al. [53]. In previous studies, immobilization, impaired feeding, sympathetic activation, inflammation and denervation were suggested to be mechanisms underlying post-stroke sarcopenia [43, 54, 55]. However, in this study, no leukocyte infiltration was observed at any time point, and only sporadic neurogenic muscle atrophy was detected at 14 days post-MCAO, which indicated that inflammation and denervation might not be the principal triggers of post-stroke sarcopenia. On account that Wall and colleagues demonstrated that rapid reduction in limb strength and muscle mass could occur during only 5 days of disuse [56] and that the diameters of bilateral gastrocnemius muscles in MCAO rats gradually increased when the neurological deficits were partially relieved and the mobility of rats increased, we suggested that post-stroke sarcopenia could be partially explained by disuse atrophy caused by hypokinesia of the limbs (hypokinesia of the paretic limb led to decreased motility of the rats, leading to disuse atrophy in the ipsilesional limb). In addition, the reduced muscle mass might partially contribute to the decreased CMAP amplitudes at 7/14 days post-MCAO. Hence, rehabilitation exercises were recommended for stroke patients as early as possible.

In this study, at 14 days after stroke, the function of the lower motor unit significantly improved, as indicated by the increased CMAP amplitudes and enlarged muscle fiber diameters, alongside the attenuated overall neurological deficits. In contrast, transsynaptic degeneration of neurons in the contralateral VH was observed, along with randomly distributed neurogenic muscle atrophy in the corresponding gastrocnemius muscles. This discrepancy implied that transsynaptic degeneration of VH neurons existed but played a minor role in lower motor system dysfunction after stroke.

The loss of motor neurons in the contralateral VH after stroke was consistent with previous papers [19, 57, 58], and the neurogenic muscle atrophy found in the corresponding hindlimb muscle in our study further offered additional proof. Compared to the reports of Dang et al. and FU et al., contralesional VH motor neuron loss occurred over a slower time course in both this study and Lin’s work, which might be attributed to different types of animal models (rats in the work of Dang et al. suffered from renovascular hypertension, which might exacerbate the process of neuron damage) and different methods to count motor neurons. Transsynaptic degeneration of contralesional VH motor neurons might partly explain the phenomena that reduced functional coupling between cortical activity and muscle output appeared early and lasted in the chronic phase where the motor function recovered greatly [59], and might also explain the condition that signs of denervation could be observed in patients with chronic stroke [13].

In this study, the proportion of p-ERK-positive motor neurons significantly increased in the contralesional VH, which paralleled the loss of motor neurons on day 14 post-MCAO. The MAPK/ERK signaling pathway, working at two different stages (throughout development and in adulthood), has pleiotropic effects in the central nervous system [26, 60–62]. During adulthood, improper activation of the ERK signaling pathway was demonstrated to exacerbate the damage in acute pathological conditions, such as ischemic stroke [63–65], traumatic brain injury [66] and spinal cord injury [67–69], to be involved in the pathogenesis of neurodegenerative diseases [26, 60, 70] and depression [71]. Hence, ERK activation might exert a detrimental effect on the transsynaptic degeneration of neurons. Moreover, the process triggered by MAPK/ERK activation partially depends on subcellular locations, as reviewed by Albert-Gascó et al. [60], and cytoplasmic p-ERK anchored by death-associated protein kinase1 (DAPK) is involved in apoptosis and neurodegeneration [64]. Indeed, the immunoreactivity of p-ERK was mainly located in the cytoplasm of motor neurons at 14 days after stroke in this study, which further suggested the harmful effect of ERK activation. In recent years, some scientists have focused on therapeutics targeting the ERK pathway in neurodegenerative diseases. NE3107, a novel small molecule targeting the ERK pathway, increased the number of surviving dopaminergic neurons in animal models of Parkinson’s disease [72], and there are currently phase III clinical trials of this drug being conducted in patients with Alzheimer’s disease [73]. We hope that the above therapeutics work well in patients with neurodegenerative diseases. In addition, we suggest that disrupting the improper activation of the ERK pathway might be a promising therapeutic target against stroke-related transsynaptic degeneration.

Although Balch and colleagues discovered convincing pathological changes following ischemic stroke in which pre- and postsynaptic maladaptation occurred at NMJs, including polyaxonal innervation [20], apparent functional abnormalities of the NMJs were not detected in this study, which indicates that maladaptation of NMJ morphology post stroke might play a minor role in the triggering and progression of lower motor functional abnormalities.

There were some limitations in the present study. First, because we aimed to explore the overall changes in the lower motor neuron system after focal ischemic stroke, in-depth exploration focusing on specific abnormalities was lacking. Moreover, this study was based on electrophysiological and pathological examinations, and further evaluation might be carried out at the transcription, translation, and conformation levels to further reveal the underlying mechanism of transsynaptic degeneration.

## Conclusions

Taken together, in this study, we demonstrated that transsynaptic degeneration of contralateral VH neurons existed during the subacute phase of focal ischemic stroke, and suggested that upregulated activation of ERK 1/2 might be involved in the initiation and progression of this pathological process. Moreover, this study offered an overview of changes in the lower motor system in MCAO rats, and we hope it will provide new information for interpreting the lower motor abnormalities post stroke to future researchers.

## Supporting information

S1 Table The mNSS scoring standards. (DOCX)

S1 File Raw data. (XLSX)

S1 Checklist *PLOS ONE* humane endpoints checklist. (DOCX)

## References

[1] Tsao CW, Aday AW, Almarzooq ZI, Anderson CAM, Arora P, Avery CL, et al. Heart Disease and Stroke Statistics-2023 Update: A Report From the American Heart Association. Circulation. 2023;147(8):e93–e621. Epub 20230125. doi: 10.1161/CIR.0000000000001123 . 36695182 PMC12135016

[2] Balch MHH, Nimjee SM, Rink C, Hannawi Y. Beyond the Brain: The Systemic Pathophysiological Response to Acute Ischemic Stroke. J Stroke. 2020;22(2):159–72. Epub 20200531. doi: 10.5853/jos.2019.02978 . 32635682 PMC7341014

[3] Sharma S, Chitranshi N, Wall RV, Basavarajappa D, Gupta V, Mirzaei M, et al. Transsynaptic degeneration in the visual pathway: Neural connectivity, pathophysiology, and clinical implications in neurodegenerative disorders. Surv Ophthalmol. 2022;67(2):411–26. Epub 20210617. doi: 10.1016/j.survophthal.2021.06.001 . 34146577

[4] Nagasawa H, Kogure K. Exo-focal postischemic neuronal death in the rat brain. Brain Res. 1990;524(2):196–202. doi: 10.1016/0006-8993(90)90690-d . 2292002

[5] Hardy J, Revesz T. The spread of neurodegenerative disease. N Engl J Med. 2012;366(22):2126–8. doi: 10.1056/NEJMcibr1202401 . 22646635

[6] Zhang J, Zhang Y, Xing S, Liang Z, Zeng J. Secondary neurodegeneration in remote regions after focal cerebral infarction: a new target for stroke management? Stroke. 2012;43(6):1700–5. Epub 20120405. doi: 10.1161/STROKEAHA.111.632448 . 22492515

[7] Geevasinga N, Menon P, Özdinler PH, Kiernan MC, Vucic S. Pathophysiological and diagnostic implications of cortical dysfunction in ALS. Nat Rev Neurol. 2016;12(11):651–61. Epub 20160923. doi: 10.1038/nrneurol.2016.140 . 27658852

[8] Suzuki H, Oyanagi K, Takahashi H, Ikuta F. Evidence for transneuronal degeneration in the spinal cord in man: a quantitative investigation of neurons in the intermediate zone after long-term amputation of the unilateral upper arm. Acta Neuropathol. 1995;89(5):464–70. doi: 10.1007/BF00307654 . 7618445

[9] Hosp JA, Greiner KL, Martinez Arellano L, Roth F, Löffler F, Reis J, et al. Progressive secondary exo-focal dopaminergic neurodegeneration occurs in not directly connected midbrain nuclei after pure motor-cortical stroke. Exp Neurol. 2020;327:113211. Epub 20200124. doi: 10.1016/j.expneurol.2020.113211 . 31987834

[10] Kondo A, Nagara H, Tateishi J. A morphometric study of myelinated fibers in the fifth lumbar ventral roots in patients with cerebrovascular diseases. Clin Neuropathol. 1987;6(6):250–6. . 3322622

[11] Terao S, Li M, Hashizume Y, Osano Y, Mitsuma T, Sobue G. Upper motor neuron lesions in stroke patients do not induce anterograde transneuronal degeneration in spinal anterior horn cells. Stroke. 1997;28(12):2553–6. doi: 10.1161/01.str.28.12.2553 . 9412648

[12] Qiu Y, Wada Y, Otomo E, Tsukagoshi H. Morphometric study of cervical anterior horn cells and pyramidal tracts in medulla oblongata and the spinal cord in patients with cerebrovascular diseases. J Neurol Sci. 1991;102(2):137–43. doi: 10.1016/0022-510x(91)90061-b . 2072114

[13] Yao B, Klein CS, Hu H, Li S, Zhou P. Motor Unit Properties of the First Dorsal Interosseous in Chronic Stroke Subjects: Concentric Needle and Single Fiber EMG Analysis. Front Physiol. 2018;9:1587. Epub 20181203. doi: 10.3389/fphys.2018.01587 . 30559674 PMC6287192

[14] Lukács M, Vécsei L, Beniczky S. Changes in muscle fiber density following a stroke. Clin Neurophysiol. 2009;120(8):1539–42. Epub 20090628. doi: 10.1016/j.clinph.2009.06.001 . 19564129

[15] Hara Y, Masakado Y, Chino N. The physiological functional loss of single thenar motor units in the stroke patients: when does it occur? Does it progress? Clin Neurophysiol. 2004;115(1):97–103. doi: 10.1016/j.clinph.2003.08.002 . 14706475

[16] Kouzi I, Trachani E, Anagnostou E, Rapidi CA, Ellul J, Sakellaropoulos GC, et al. Motor unit number estimation and quantitative needle electromyography in stroke patients. J Electromyogr Kinesiol. 2014;24(6):910–6. Epub 20140928. doi: 10.1016/j.jelekin.2014.09.006 . 25304197

[17] Li X, Fisher M, Rymer WZ, Zhou P. Application of the F-Response for Estimating Motor Unit Number and Amplitude Distribution in Hand Muscles of Stroke Survivors. IEEE Trans Neural Syst Rehabil Eng. 2016;24(6):674–81. Epub 20150708. doi: 10.1109/TNSRE.2015.2453274 . 26168437 PMC4902775

[18] Arasaki K, Igarashi O, Ichikawa Y, Machida T, Shirozu I, Hyodo A, et al. Reduction in the motor unit number estimate (MUNE) after cerebral infarction. J Neurol Sci. 2006;250(1–2):27–32. Epub 20060809. doi: 10.1016/j.jns.2006.06.024 . 16904126

[19] Lin N, Liu MS, Fan SY, Guan YZ, Cui LY. Asynchronization in Changes of Electrophysiology and Pathology of Spinal Cord Motor Neurons in Rats Following Middle Cerebral Artery Occlusion. Chin Med J (Engl). 2015;128(21):2919–25. doi: 10.4103/0366-6999.168057 . 26521791 PMC4756893

[20] Balch MHH, Harris H, Chugh D, Gnyawali S, Rink C, Nimjee SM, et al. Ischemic stroke-induced polyaxonal innervation at the neuromuscular junction is attenuated by robot-assisted mechanical therapy. Exp Neurol. 2021;343:113767. Epub 20210525. doi: 10.1016/j.expneurol.2021.113767 . 34044000 PMC8286354

[21] Schmitt AB, Brook GA, Buss A, Nacimiento W, Noth J, Kreutzberg GW. Dynamics of microglial activation in the spinal cord after cerebral infarction are revealed by expression of MHC class II antigen. Neuropathol Appl Neurobiol. 1998;24(3):167–76. doi: 10.1046/j.1365-2990.1998.00103.x . 9717181

[22] Wu YP, Ling EA. Induction of microglial and astrocytic response in the adult rat lumbar spinal cord following middle cerebral artery occlusion. Exp Brain Res. 1998;118(2):235–42. doi: 10.1007/s002210050277 . 9547093

[23] Dihné M, Grommes C, Lutzenburg M, Witte OW, Block F. Different mechanisms of secondary neuronal damage in thalamic nuclei after focal cerebral ischemia in rats. Stroke. 2002;33(12):3006–11. doi: 10.1161/01.str.0000039406.64644.cb . 12468804

[24] Stuckey SM, Ong LK, Collins-Praino LE, Turner RJ. Neuroinflammation as a Key Driver of Secondary Neurodegeneration Following Stroke? Int J Mol Sci. 2021;22(23). Epub 20211203. doi: 10.3390/ijms222313101 . 34884906 PMC8658328

[25] Kirouac L, Rajic AJ, Cribbs DH, Padmanabhan J. Activation of Ras-ERK Signaling and GSK-3 by Amyloid Precursor Protein and Amyloid Beta Facilitates Neurodegeneration in Alzheimer’s Disease. eNeuro. 2017;4(2). Epub 20170327. doi: 10.1523/ENEURO.0149-16.2017 . 28374012 PMC5367084

[26] Rai SN, Dilnashin H, Birla H, Singh SS, Zahra W, Rathore AS, et al. The Role of PI3K/Akt and ERK in Neurodegenerative Disorders. Neurotox Res. 2019;35(3):775–95. Epub 20190201. doi: 10.1007/s12640-019-0003-y . 30707354

[27] Sahu R, Upadhayay S, Mehan S. Inhibition of extracellular regulated kinase (ERK)-1/2 signaling pathway in the prevention of ALS: Target inhibitors and influences on neurological dysfunctions. Eur J Cell Biol. 2021;100(7–8):151179. Epub 20210917. doi: 10.1016/j.ejcb.2021.151179 . 34560374

[28] Xu Z, Wang BR, Wang X, Kuang F, Duan XL, Jiao XY, et al. ERK1/2 and p38 mitogen-activated protein kinase mediate iNOS-induced spinal neuron degeneration after acute traumatic spinal cord injury. Life Sci. 2006;79(20):1895–905. Epub 20060621. doi: 10.1016/j.lfs.2006.06.023 . 16978658

[29] Longa EZ, Weinstein PR, Carlson S, Cummins R. Reversible middle cerebral artery occlusion without craniectomy in rats. Stroke. 1989;20(1):84–91. doi: 10.1161/01.str.20.1.84 . 2643202

[30] Chen J, Sanberg PR, Li Y, Wang L, Lu M, Willing AE, et al. Intravenous administration of human umbilical cord blood reduces behavioral deficits after stroke in rats. Stroke. 2001;32(11):2682–8. doi: 10.1161/hs1101.098367 . 11692034

[31] Rao SD, Banack SA, Cox PA, Weiss JH. BMAA selectively injures motor neurons via AMPA/kainate receptor activation. Exp Neurol. 2006;201(1):244–52. Epub 20060609. doi: 10.1016/j.expneurol.2006.04.017 . 16764863

[32] Wu SH, Huang SH, Cheng KI, Chai CY, Yeh JL, Wu TC, et al. Third-degree hindpaw burn injury induced apoptosis of lumbar spinal cord ventral horn motor neurons and sciatic nerve and muscle atrophy in rats. Biomed Res Int. 2015;2015:372819. Epub 20150128. doi: 10.1155/2015/372819 . 25695065 PMC4324890

[33] Yin HZ, Tang DT, Weiss JH. Intrathecal infusion of a Ca(2+)-permeable AMPA channel blocker slows loss of both motor neurons and of the astrocyte glutamate transporter, GLT-1 in a mutant SOD1 rat model of ALS. Exp Neurol. 2007;207(2):177–85. Epub 20070724. doi: 10.1016/j.expneurol.2007.07.011 . 17719032 PMC2083564

[34] Stephens B, Guiloff RJ, Navarrete R, Newman P, Nikhar N, Lewis P. Widespread loss of neuronal populations in the spinal ventral horn in sporadic motor neuron disease. A morphometric study. J Neurol Sci. 2006;244(1–2):41–58. Epub 20060217. doi: 10.1016/j.jns.2005.12.003 . 16487542

[35] Lin W, Li M, Li Y, Sun X, Li X, Yang F, et al. Bone marrow stromal cells promote neurite outgrowth of spinal motor neurons by means of neurotrophic factors in vitro. Neurol Sci. 2014;35(3):449–57. Epub 20130706. doi: 10.1007/s10072-013-1490-x . 23832111

[36] Alvarez S, Moldovan M, Krarup C. Prolonged high frequency electrical stimulation is lethal to motor axons of mice heterozygously deficient for the myelin protein P₀ gene. Exp Neurol. 2013;247:552–61. Epub 20130222. doi: 10.1016/j.expneurol.2013.02.006 . 23439028

[37] Kim MG, Lim H, Lee HS, Han IJ, Ku J, Kang YJ. Brain-computer interface-based action observation combined with peripheral electrical stimulation enhances corticospinal excitability in healthy subjects and stroke patients. J Neural Eng. 2022;19(3). Epub 20220620. doi: 10.1088/1741-2552/ac76e0 . 35675795

[38] de Freitas Zanona A, Romeiro da Silva AC, do Rego Maciel AB, Gomes do Nascimento LS, Bezerra da Silva A, Bolognini N, et al. Somatosensory Cortex Repetitive Transcranial Magnetic Stimulation and Associative Sensory Stimulation of Peripheral Nerves Could Assist Motor and Sensory Recovery After Stroke. Front Hum Neurosci. 2022;16:860965. Epub 20220411. doi: 10.3389/fnhum.2022.860965 . 35479184 PMC9036089

[39] Huang L, Li M, Deng C, Qiu J, Wang K, Chang M, et al. Potential Therapeutic Strategies for Skeletal Muscle Atrophy. Antioxidants (Basel). 2022;12(1). Epub 20221226. doi: 10.3390/antiox12010044 . 36670909 PMC9854691

[40] Global, regional, and national burden of stroke, 1990–2016: a systematic analysis for the Global Burden of Disease Study 2016. Lancet Neurol. 2019;18(5):439–58. Epub 20190311. doi: 10.1016/S1474-4422(19)30034-1 . 30871944 PMC6494974

[41] Gorelick PB. The global burden of stroke: persistent and disabling. Lancet Neurol. 2019;18(5):417–8. Epub 20190311. doi: 10.1016/S1474-4422(19)30030-4 . 30871943

[42] Huang C, Yao B, Li X, Li S, Zhou P. Muscle Fiber Diameter and Density Alterations after Stroke Examined by Single-Fiber EMG. Neural Plast. 2021;2021:3045990. Epub 20210813. doi: 10.1155/2021/3045990 . 34434227 PMC8380495

[43] Li W, Yue T, Liu Y. New understanding of the pathogenesis and treatment of stroke-related sarcopenia. Biomed Pharmacother. 2020;131:110721. Epub 20200910. doi: 10.1016/j.biopha.2020.110721 . 32920517

[44] Wu YP, Ling EA. Transsynaptic changes of neurons and associated microglial reaction in the spinal cord of rats following middle cerebral artery occlusion. Neurosci Lett. 1998;256(1):41–4. doi: 10.1016/s0304-3940(98)00750-2 . 9832212

[45] Umpierre AD, Wu LJ. How microglia sense and regulate neuronal activity. Glia. 2021;69(7):1637–53. Epub 20201228. doi: 10.1002/glia.23961 . 33369790 PMC8113084

[46] Badimon A, Strasburger HJ, Ayata P, Chen X, Nair A, Ikegami A, et al. Negative feedback control of neuronal activity by microglia. Nature. 2020;586(7829):417–23. Epub 20200930. doi: 10.1038/s41586-020-2777-8 . 32999463 PMC7577179

[47] Eyo UB, Peng J, Swiatkowski P, Mukherjee A, Bispo A, Wu LJ. Neuronal hyperactivity recruits microglial processes via neuronal NMDA receptors and microglial P2Y12 receptors after status epilepticus. J Neurosci. 2014;34(32):10528–40. doi: 10.1523/JNEUROSCI.0416-14.2014 . 25100587 PMC4200107

[48] Cserép C, Pósfai B, Lénárt N, Fekete R, László ZI, Lele Z, et al. Microglia monitor and protect neuronal function through specialized somatic purinergic junctions. Science. 2020;367(6477):528–37. Epub 20191212. doi: 10.1126/science.aax6752 . 31831638

[49] Dong Y, D’Mello C, Pinsky W, Lozinski BM, Kaushik DK, Ghorbani S, et al. Oxidized phosphatidylcholines found in multiple sclerosis lesions mediate neurodegeneration and are neutralized by microglia. Nat Neurosci. 2021;24(4):489–503. Epub 20210218. doi: 10.1038/s41593-021-00801-z . 33603230

[50] Schubert P, Ogata T, Marchini C, Ferroni S, Rudolphi K. Protective mechanisms of adenosine in neurons and glial cells. Ann N Y Acad Sci. 1997;825:1–10. doi: 10.1111/j.1749-6632.1997.tb48409.x . 9369970

[51] Wu YP, McRae A, Rudolphi K, Ling EA. Propentofylline attenuates microglial reaction in the rat spinal cord induced by middle cerebral artery occlusion. Neurosci Lett. 1999;260(1):17–20. doi: 10.1016/s0304-3940(98)00941-0 . 10027689

[52] BathPM, Bath-HextallFJ. Pentoxifylline, propentofylline and pentifylline for acute ischaemic stroke. Cochrane Database Syst Rev. 2004;(3):Cd000162. doi: 10.1002/14651858.CD000162.pub2 .15266424

[53] English C, McLennan H, Thoirs K, Coates A, Bernhardt J. Loss of skeletal muscle mass after stroke: a systematic review. Int J Stroke. 2010;5(5):395–402. doi: 10.1111/j.1747-4949.2010.00467.x . 20854624

[54] Li S, Gonzalez-Buonomo J, Ghuman J, Huang X, Malik A, Yozbatiran N, et al. Aging after stroke: how to define post-stroke sarcopenia and what are its risk factors? Eur J Phys Rehabil Med. 2022;58(5):683–92. Epub 20220905. doi: 10.23736/S1973-9087.22.07514-1 . 36062331 PMC10022455

[55] Yoshimura Y, Wakabayashi H, Bise T, Tanoue M. Prevalence of sarcopenia and its association with activities of daily living and dysphagia in convalescent rehabilitation ward inpatients. Clin Nutr. 2018;37(6 Pt A):2022–8. Epub 20170923. doi: 10.1016/j.clnu.2017.09.009 . 28987469

[56] Wall BT, Dirks ML, Snijders T, Senden JM, Dolmans J, van Loon LJ. Substantial skeletal muscle loss occurs during only 5 days of disuse. Acta Physiol (Oxf). 2014;210(3):600–11. Epub 20131205. doi: 10.1111/apha.12190 . 24168489

[57] Dang G, Chen X, Chen Y, Zhao Y, Ouyang F, Zeng J. Dynamic secondary degeneration in the spinal cord and ventral root after a focal cerebral infarction among hypertensive rats. Sci Rep. 2016;6:22655. Epub 20160307. doi: 10.1038/srep22655 . 26949108 PMC4780069

[58] Fu D, Ng YK, Gan P, Ling EA. Permanent occlusion of the middle cerebral artery upregulates expression of cytokines and neuronal nitric oxide synthase in the spinal cord and urinary bladder in the adult rat. Neuroscience. 2004;125(4):819–31. doi: 10.1016/j.neuroscience.2004.02.012 . 15120843

[59] Larsen LH, Zibrandtsen IC, Wienecke T, Kjaer TW, Christensen MS, Nielsen JB, et al. Corticomuscular coherence in the acute and subacute phase after stroke. Clin Neurophysiol. 2017;128(11):2217–26. Epub 20170922. doi: 10.1016/j.clinph.2017.08.033 . 28987993

[60] Albert-Gascó H, Ros-Bernal F, Castillo-Gómez E, Olucha-Bordonau FE. MAP/ERK Signaling in Developing Cognitive and Emotional Function and Its Effect on Pathological and Neurodegenerative Processes. Int J Mol Sci. 2020;21(12). Epub 20200623. doi: 10.3390/ijms21124471 . 32586047 PMC7352860

[61] Li S, Mattar P, Dixit R, Lawn SO, Wilkinson G, Kinch C, et al. RAS/ERK signaling controls proneural genetic programs in cortical development and gliomagenesis. J Neurosci. 2014;34(6):2169–90. doi: 10.1523/JNEUROSCI.4077-13.2014 . 24501358 PMC6608536

[62] Nuttall JR, Oteiza PI. Zinc and the ERK kinases in the developing brain. Neurotox Res. 2012;21(1):128–41. Epub 20111118. doi: 10.1007/s12640-011-9291-6 . 22095091 PMC4316815

[63] Maddahi A, Edvinsson L. Cerebral ischemia induces microvascular pro-inflammatory cytokine expression via the MEK/ERK pathway. J Neuroinflammation. 2010;7:14. Epub 20100226. doi: 10.1186/1742-2094-7-14 . 20187933 PMC2837637

[64] Xiong W, Wu Y, Xian W, Song L, Hu L, Pan S, et al. DAPK1-ERK signal mediates oxygen glucose deprivation reperfusion induced apoptosis in mouse N2a cells. J Neurol Sci. 2018;387:210–9. Epub 20180104. doi: 10.1016/j.jns.2018.01.003 . 29571866

[65] Qin C, Yang S, Chu YH, Zhang H, Pang XW, Chen L, et al. Signaling pathways involved in ischemic stroke: molecular mechanisms and therapeutic interventions. Signal Transduct Target Ther. 2022;7(1):215. Epub 20220706. doi: 10.1038/s41392-022-01064-1 . 35794095 PMC9259607

[66] Bhowmick S, D’Mello V, Abdul-Muneer PM. Synergistic Inhibition of ERK1/2 and JNK, Not p38, Phosphorylation Ameliorates Neuronal Damages After Traumatic Brain Injury. Mol Neurobiol. 2019;56(2):1124–36. Epub 20180606. doi: 10.1007/s12035-018-1132-7 . 29873042

[67] Abbaszadeh F, Fakhri S, Khan H. Targeting apoptosis and autophagy following spinal cord injury: Therapeutic approaches to polyphenols and candidate phytochemicals. Pharmacol Res. 2020;160:105069. Epub 20200708. doi: 10.1016/j.phrs.2020.105069 32652198

[68] Wang B, Liu S, Fan B, Xu X, Chen Y, Lu R, et al. PKM2 is involved in neuropathic pain by regulating ERK and STAT3 activation in rat spinal cord. J Headache Pain. 2018;19(1):7. Epub 20180118. doi: 10.1186/s10194-018-0836-4 . 29349661 PMC5773456

[69] Lee JY, Choi HY, Ju BG, Yune TY. Estrogen alleviates neuropathic pain induced after spinal cord injury by inhibiting microglia and astrocyte activation. Biochim Biophys Acta Mol Basis Dis. 2018;1864(7):2472–80. Epub 20180416. doi: 10.1016/j.bbadis.2018.04.006 . 29653184

[70] Muraleva NA, Kolosova NG, Stefanova NA. MEK1/2-ERK Pathway Alterations as a Therapeutic Target in Sporadic Alzheimer’s Disease: A Study in Senescence-Accelerated OXYS Rats. Antioxidants (Basel). 2021;10(7). Epub 20210630. doi: 10.3390/antiox10071058 . 34208998 PMC8300733

[71] Wang JQ, Mao L. The ERK Pathway: Molecular Mechanisms and Treatment of Depression. Mol Neurobiol. 2019;56(9):6197–205. Epub 20190209. doi: 10.1007/s12035-019-1524-3 . 30737641 PMC6684449

[72] Nicoletti F, Philippens I, Fagone P, Ahlem CN, Reading CL, Frincke JM, et al. 17α-Ethynyl-androst-5-ene-3β,7β,17β-triol (HE3286) Is Neuroprotective and Reduces Motor Impairment and Neuroinflammation in a Murine MPTP Model of Parkinson’s Disease. Parkinsons Dis. 2012;2012:969418. Epub 20120926. doi: 10.1155/2012/969418 . 23050197 PMC3463191

[73] Reading CL, Ahlem CN, Murphy MF. NM101 Phase III study of NE3107 in Alzheimer’s disease: rationale, design and therapeutic modulation of neuroinflammation and insulin resistance. Neurodegener Dis Manag. 2021;11(4):289–98. Epub 20210712. doi: 10.2217/nmt-2021-0022 . 34251287

