# Supplementary material for: Transsynaptic degeneration of ventral horn motor neurons exists but plays a minor role in lower motor system dysfunction in acute ischemic rats
Source: PLoS One. 2024 Apr 26;19(4):e0298006. doi: 10.1371/journal.pone.0298006 (PMC11051614; doi:10.1371/journal.pone.0298006)
Supplement: S1 Table — (DOCX) [file pone.0298006.s001.docx]

| Scoring sections | Scoring Standards | Points |
| --- | --- | --- |
| **Motor tests** | Raising the rat by the tail | 3 |
|  | Flexion of the forelimb | 1 |
|  | Flexion of the hindlimb | 1 |
|  | Head moving > 10° to vertical axis within 30 s | 1 |
|  | Placing the rat on the floor | 3 |
|  | Normal walk | 0 |
|  | Inability to walk straight | 1 |
|  | Circling toward paretic side | 2 |
|  | Falling down to paretic side | 3 |
| **Sensory tests** | Limb placing test (moving the rat laterally toward the table) |  |
|  | Reaching the table slowly with limbs or could not move at all | 1 |
|  | Proprioceptive test (pushing the paw against table edge to stimulate limb muscles) |  |
|  | Losing the resistance | 1 |
| **Balance tests** | Beam balance tests (2.5cm wide) | 6 |
|  | Balancing with steady posture | 0 |
|  | Grasping side of the beam | 1 |
|  | Hugging the beam and 1 limb falling down from the beam | 2 |
|  | Hugging the beam and 2 limbs falling down from beam, or spins on beam (> 60 s) | 3 |
|  | Attempting to balance on the beam but falling off (> 40 s) | 4 |
|  | Attempting to balance on the beam but falling off (> 20 s) | 5 |
|  | Falls off; no attempt to balance or hang on to beam (< 20 s) | 6 |
| **Reflexes** | Touching the auditory meatus |  |
|  | Not shaking the head | 1 |
|  | Slightly touching the cornea with cotton |  |
|  | No eye blink | 1 |
|  | Making a brief noise |  |
|  | No motor response | 1 |
| **Abnormal movements** | Seizures, myoclonus, or myodystony | 1 |
| **Maximum points** |  | 18 |

**Supplementary table 1.** The modified neurological severity score scoring standards.
